# Supplementary material for: Stability Engineering of Recombinant Secretory IgA
Source: Int J Mol Sci. 2024 Jun 22;25(13):6856. doi: 10.3390/ijms25136856 (PMC11240955; doi:10.3390/ijms25136856)
Supplement: Supplementary file 1 [file ijms-25-06856-s001.zip › ijms-3018685-supplementary.pdf]

Supplemental Figures

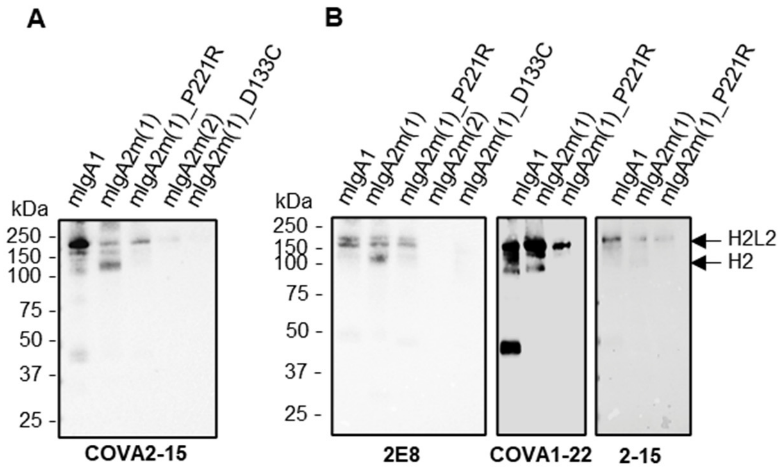

Figure S1: Immunoblotting of monomeric κLC (A) and λLC (B) IgA variants. SDS-PAGE under non-reducing conditions of crude leaf extracts from *N. benthamiana* ΔXT/FT infiltrated with different mIgA variants. After blotting bands were visualized using an HRP-labeled anti-IgA-HC antibody.

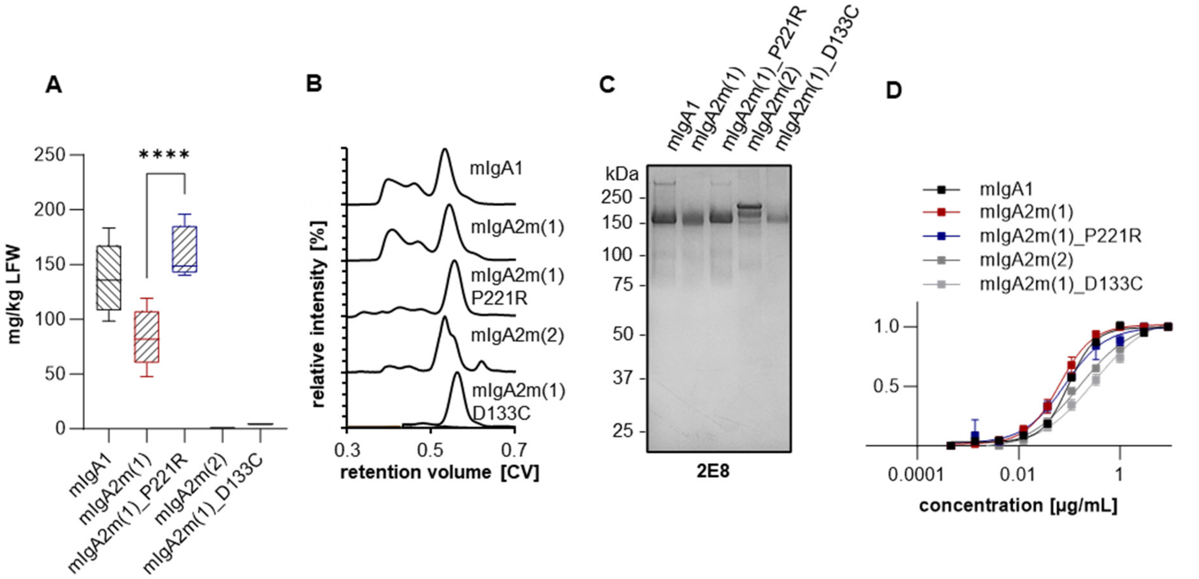

Figure S2: Expression and characterization of stability engineered monomeric 2E8 IgA variants. (A) Monomeric IgA1 and IgA2 recognizing the SARS-CoV-2 spike protein were transiently expressed in *Nicotiana benthamiana* ΔXT/FT plants. Expression levels were quantified by sandwich ELISA in crude leaf extracts. Detection of monomeric IgA was with anti-lambda light chain antibodies. Quantification data represent the mean of two technical repeats of three independent infiltrations of 3 plants each ± SD. One-way ANOVA was performed to compare the groups;~~ **p < 0.01; ***p < 0.001; ~~ ****p < 0.0001 (**B**) Normalized size-exclusion chromatograms of affinity-purified monomeric IgA isotypes from infiltrated *N. benthamiana* ΔXT/FT leaves. Curves are representatives of two individual runs with similar results. Values were normalized based on the highest signal of each chromatogram. (**C**) SDS-PAGE under non-reducing conditions of affinity and size-exclusion purified plant-produced monomeric IgA1/IgA2 visualized by Coomassie Brilliant Blue staining. (**D**) Determination of EC_{50} values of monomeric IgA variants to the receptor binding domain (RBD) of the SARS-CoV-2 spike protein. Each value is the mean ± SD from three independent measurements.

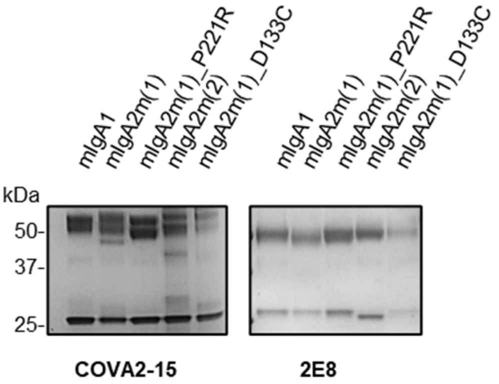

Figure S3: SDS-PAGE of COVA2-15 and 2E8 monomeric IgA variants. SDS-PAGE was run under reducing conditions of affinity and size-exclusion purified plant-produced monomeric IgA1/IgA2 visualized by Coomassie Brilliant Blue staining.

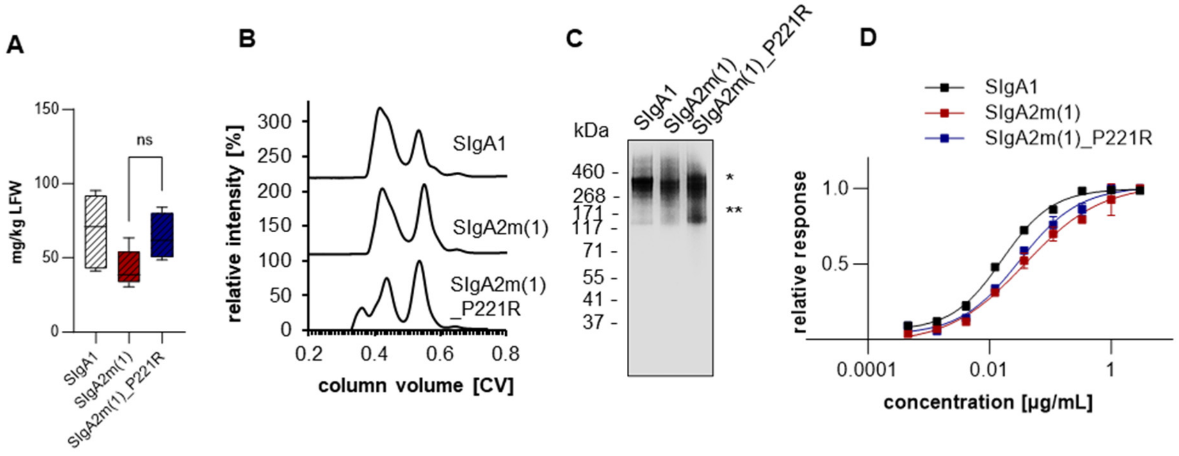

Figure S4: Expression and characterization of stability engineered 2E8 SIgA. (A) Secretory IgA1 and IgA2 recognizing the SARS-CoV-2 spike protein were transiently expressed in *N. benthamiana* ΔXT/FT plants. Expression levels were quantified by sandwich ELISA in crude leaf extracts. Secretory IgA antibodies were detected using anti-secretory component antibodies. Quantification data represent the mean of two technical repeats of three independent infiltrations of 3 plants each ± SD. One-way ANOVA was performed to compare the groups; ~~**p < 0.01; ***p < 0.001; ****p < 0.0001;~~ ns: not significant; (**B**) Normalized size-exclusion chromatograms of affinity-purified monomeric IgA isotypes from infiltrated *N. benthamiana* ΔXT/FT leaves. Curves are representatives of two individual runs with similar results. Values were normalized based on the highest signal of each chromatogram. (**C**) SDS-PAGE under non-reducing conditions of affinity and size-exclusion purified plant-produced secretory IgA1/IgA2 visualized by Coomassie Brilliant Blue staining; fully assembled SIgA (*), monomeric IgA (**). (**D**) Determination of EC_{50} values of secretory IgA variants to the receptor binding domain (RBD) of the SARS-CoV-2 spike protein. Each value is the mean ± SD from three independent measurements.

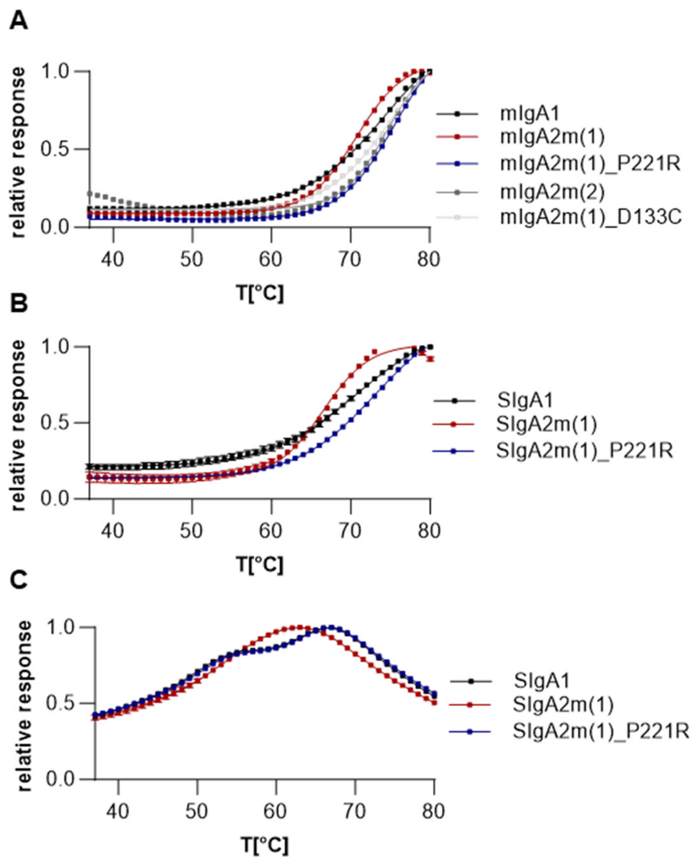

Figure S5: Thermal unfolding of 2E8 monomeric and secretory IgA. Differential scanning fluorimetry curves of monomeric (**A**) and secretory (**B**) IgA isotypes in 1xPBS buffer pH 7.4 or citrate buffer pH 3.5 (**C**). Experiments were performed at 1mg/mL. DSF curves are the mean of three technical repeats of one out of three independent experiments with similar outcome.

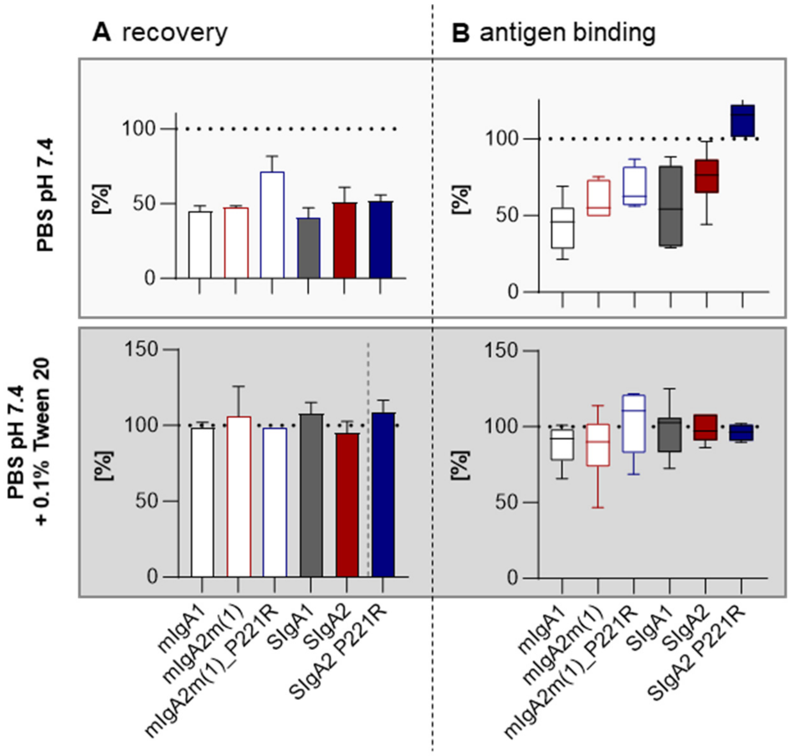

Figure S6: Aerosolization of 2E8 IgA antibodies using the Omron MicroAir U22 portable mesh nebulizer. 1 mL of monoclonal antibody with a concentration of 500 µg/mL was nebulized and condensate was collected. (**A**) Protein concentration in the condensate after aerosolization was measured using absorbance at 280 nm. (**B**) Antigen binding of samples before and after nebulization were tested by ELISA with equal protein loading. Values for recovery and antigen binding activity are means ± SD of at least three independent aerosolization experiments.

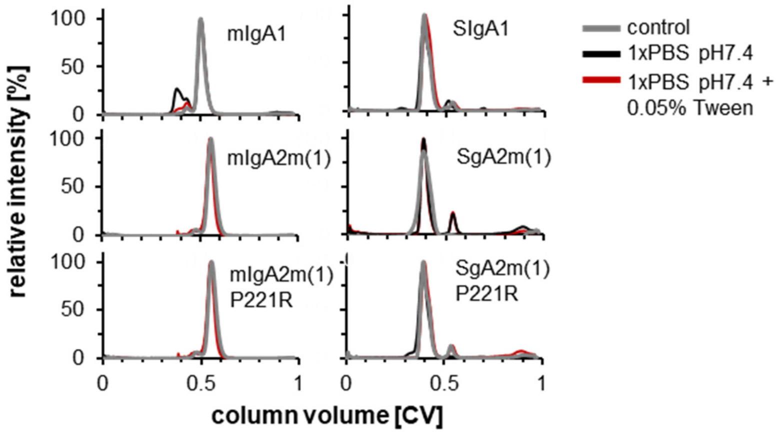

Figure S7: Size-exclusion chromatograms of COVA2-15 IgA monoclonal antibodies before and after nebulization. Normalized size-exclusion chromatograms of affinity and size-exclusion purified monomeric and secretory IgA isotypes from infiltrated *N. benthamiana* ΔXT/FT leaves before (grey) and after nebulization formulated in either PBS (black) or PBS-T (red). Curves are representatives of two individual runs with similar results. Values were normalized based on the highest signal of each chromatogram.

Supplemental Tables

Table S1: Purification yields of COVA2-15 and 2E8 IgAs. Yields after affinity purification and size exclusion chromatography from 100 g *N. benthamiana* leaves were determined by measurements of absorbance at 280 nm (A_{280}).

		mg/kg LFW
COVA2-15	mIgA1	81.9
	mIgA2m(1)	69.8
	mIgA2m(1)_P221R	129.2
	mIgA2m(2)	4.9
	mIgA2m(1)_D133C	3.3
	SIgA1	35.4
	SIgA2m(1)	25.6
	SIgA2m(1)_P221R	55.0
2E8	mIgA1	35.4
	mIgA2m(1)	20.0
	mIgA2m(1)_P221R	50.7
	mIgA2m(2)	2.9
	mIgA2m(1)_D133C	0.9
	SIgA1	20.2
	SIgA2m(1)	15.1
	SIgA2m(1)_P221R	18.6

Table S2: EC50 values of mIgA binding to antigen RBD.

	mAb	EC_{50} [ng/mL]		
COVA2-15	mIgA1	9.25	±	0.82
	mIgA2m(1)	5.98	±	0.63
	mIgA2m(1)_P221R	3.03	±	0.73
	mIgA2m(2)	16.16	±	0.24
	mIgA2m(1)_D133C	55.92	±	1.59
2E8	mIgA1	96.54	±	1.15
	mIgA2m(1)	63.84	±	1.20
	mIgA2m(1)_P221R	80.69	±	5.19
	mIgA2m(2)	163.30	±	1.38
	mIgA2m(1)_D133C	306.70	±	2.14

Table S3: EC50 values of SIgA binding to antigen RBD.

	mAb	EC_{50} [ng/mL]
COVA2-15	SIgA1	6.46 ± 0.42
	SIgA2m(1)	6.67 ± 0.79
	SIgA2m(1)_P221R	8.18 ± 0.17
2E8	SIgA1	16.15 ± 0.69
	SIgA2m(1)	37.94 ± 2.71
	SIgA2m(1)_P221R	30.94 ± 0.42

Table S4: Midpoint temperatures of thermal unfolding (T_m) of 2E8 monomeric and secretory IgA mutants at physiological conditions. Thermal shift assays were performed in 1xPBS buffer pH 7.4. Values represent the median of at least three independent differential scanning fluorimetry experiments with 3 repeats each ±SD.

	T_m [°C]
mIgA1	74.5 ± 0.9
mIgA2	71.4 ± 0.5
mIgA2m(1)_P221R	75.4 ± 0.3
mIgA2m(2)	76.5 ± 2.6
mIgA2m(1)_D133C	75.7 ± 0.5
SIgA1	71.4 ± 0.9
SIgA2m(1)	68.2 ± 1.4
SIgA2m(1)_P221R	72.6 ± 2.4

Table S5: Midpoint temperatures of thermal unfolding (T_m) of 2E8 secretory IgA mutants in acidic conditions. Thermal shift assays were performed in citrate buffer pH 3.5. Values represent the median of three independent differential scanning fluorimetry experiments with 3 repeats each ±SD.

	2E8	
	T_{m1} [°C]	T_{m2} [°C]
SIgA1	49.8 ± 0.4	63.5 ± 0.0
SIgA2m(1)	53.4 ± 0.4	/
SIgA2m(1)_P221R	50.5 ± 0.0	63.4 ± 0.2

Table S6: Recovery and antigen binding capacity of mAbs in the aersolisation condensate

	mAb	recovery [%]		antigen binding [%]	
		PBS	PBS-T	PBS	PBS-T
COVA2-15	**IgG**	70.9 ± 14.9	103.5 ± 7.4	66.6 ± 21.2	103.0 ± 24.9
	**mIgA1**	67.7 ± 14.0	102.2 ± 7.8	55.0 ± 13.1	84.4 ± 10.4
	**mIgA2m(1)**	60.0 ± 12.0	111.3 ± 5.6	62.2 ± 13.9	89.6 ± 15.9
	**mIgA2m(1)_P221R**	75.0 ± 3.2	97.6 ± 0.3	108.3 ± 8.3	91.2 ± 11.1
	**SIgA1**	37.2 ± 1.7	111.0 ± 12.8	62.4 ± 11.5	94.3 ± 7.1
	**SIgA2**	32.9 ± 1.4	100.0 ± 10.1	53.3 ± 12.3	106.8 ± 14.0
	**SIgA2 P221R**	46.6 ± 7.6	112.0 ± 19.2	89.4 ± 9.9	111.3 ± 14.5
2E8	**IgG**	70.4 ± 2.0	83.9 ± 12.9	36.9 ± 9.5	114.1 ± 14.1
	**mIgA1**	44.6 ± 4.0	98.4 ± 3.5	34.4 ± 14.0	88.5 ± 12.3
	**mIgA2m(1)**	47.6 ± 1.0	105.9 ± 19.8	59.6 ± 11.7	86.4 ± 19.8
	**mIgA2m(1)_P221R**	71.6 ± 10.2	98.3 ± 0.0	67.0 ± 14.1	103.7 ± 22.0
	**SIgA1**	40.4 ± 6.8	107.9 ± 7.3	56.1 ± 24.7	98.3 ± 14.5
	**SIgA2**	50.9 ± 10.2	95.4 ± 7.3	79.9 ± 10.8	97.7 ± 8.4
	**SIgA2 P221R**	52.0 ± 4.0	109.0 ± 7.7	112.7 ± 11.7	96.2 ± 5.7

Table S7: Parameters determined by dynamic light scattering of COVA2-15 SIgA2m(1) and SIgA2m(1)_P221R before and after aerosolization using the Omron MicroAir U22 portable mesh nebulizer. The mean diameter and the homogeneity were measured by intensity or volume using a Malvern Zetasizer nano-ZS (Malvern Instruments Ltd., Worcestershire, UK) at 25°C. Each sample was measured in triplicates.

			Intesity				Volume	
**sample**	**formulation**	**PdI**	**Pk1**	**Pk2**	**Pk1**	**Pk2**	**Pk1**	**Pk2**
			[nm]		[%]		[%]	
SIgA2m(1)	1xPBS	0.214	36.6 ± 18.4		100.0		100.0	
SIgA2m(1) condensate	1xPBS	0.380	25.3 ± 7.5	106.8 ± 57.9	71.2	28.8	99.7	0.3
SIgA2m(1) condensate	1xPBS-T 0.05%	0.527	34.1 ± 22.6	278.9 ± 125.0	86.4	13.6	99.9	0.1
SIgA2m(1)_P221R	1xPBS	0.165	28.6 ± 10.5	±	100.0		100.0	
SIgA2m(1)_P221R condensate	1xPBS	0.369	26.5 ± 8.9	353.0 ± 140.7	80.1	19.5	99.8	0.2
SIgA2m(1)_P221R condensate	1xPBS-T 0.05%	0.344	29.0 ± 13.9	327.0 ± 143.1	89.6	10.4	99.9	0.1

Table S8: Primers used for site-directed mutagenesis (SDM)

	**'5-3' sequence**
**IgA2m(1)_D133C-fw**	GTTTTCCCACTCTCCCTCtgtTCTACTCCACA
**IgA2m(1)_D133C-rev**	TGTGGAGTAGAacaGAGGGAGAGTGGGAAAAC
**IgA2m(1)_P221R-fw**	TGTTCCATGTAGAGTTCCACCAC
**IgA2m(1)_P221R-rev**	GTAACATCCTGGGATGGGTTAGT
**IgA2m(1)_P212S-fw**	CACTACACTAACAGCTCCCAGGATGTTAC
**IgA2m(1)_P212S-rev**	GTAACATCCTGGGAGCTGTTAGTGTAGTG
